# Crystal structure of *N*,*N*′-bis­(pyridin-4-ylmeth­yl)cyclo­hexane-1,4-di­ammonium dichloride dihydrate

**DOI:** 10.1107/S2056989016014626

**Published:** 2016-09-23

**Authors:** Suk-Hee Moon, Donghyun Kang, Ki-Min Park

**Affiliations:** a Department of Food and Nutrition, Kyungnam College of Information and Technology, Busan 47011, Republic of Korea; b Department of Science Education, Kyungnam University, Changwon 51767, Republic of Korea; c Research institute of Natural Science, Gyeongsang National University, Jinju 52828, Republic of Korea

**Keywords:** crystal structure, diprotonated structure, dipyridyl salt, hydrogen bonding, condensation reaction.

## Abstract

Treatment of *N*,*N*-bis­(pyridin-4-ylmeth­yl)cyclo­hexane-1,4-di­amine with hydro­chloric acid in ethanol led to the formation of the title salt, C_18_H_26_N_4_^2+^·2Cl^−^·2H_2_O, which lies about a crystallographic inversion center at the center of the cyclo­hexyl ring. The asymmetric unit therefore comprises one half of the *N*,*N*-bis­(pyridin-4-ylmeth­yl)cyclo­hexane-1,4-di­ammonium dication, a chloride anion, and a solvent water mol­ecule. In the dication, the two *trans*-(4-pyridine)–CH_2_–NH_2_– moieties occupy equatorial sites at the 1- and 4-positions of the central cyclo­hexyl ring, which is in a chair conformation. The terminal pyridine ring is tilted by 27.98 (5)° with respect to the mean plane of the central cyclo­hexyl moiety (r.m.s. deviation = 0.2379 Å). In the crystal, dications, anions, and solvent water mol­ecules are connected *via* N/C/O—H⋯Cl and N—H⋯O hydrogen bonds together with C—H⋯π inter­actions, forming a three-dimensional network.

## Abstract 2

In the title salt, C_18_H_26_N_4_^2+^2·Cl^−^·2H_2_O, the *N*,*N*-bis­(pyridin-4-ylmeth­yl)cyclo­hexane-1,4-di­ammonium dication lies on a crystallographic inversion center, and the central cyclo­hexyl ring adopts a chair conformation. In the crystal, dications, anions and solvent water mol­ecules are connected *via* N/C/O—H⋯Cl and N—H⋯O hydrogen bonds and C—H⋯π inter­actions, forming a three-dimensional network.

## Chemical context   

An enormous number of metal–organic frameworks (MOFs) have been developed over the last two decades because of their attractive topologies and their desirable applications in a wide range of fields (Silva *et al.*, 2015 ▸; Furukawa *et al.*, 2014 ▸). For the development of these MOFs, many chemists have designed and prepared various dipyridyl-type ligands (Robin & Fromm, 2006 ▸; Robson, 2008 ▸; Leong & Vittal, 2011 ▸). Our group has also focused on the search for extended dipyridyl-type ligands with a bulky central section for the development of MOFs with intriguing topologies or useful properties. As a part of our ongoing efforts, we prepared just such a dipyridyl-type ligand with a central cyclo­hexyl moiety, namely *N*,*N*-bis­(pyridin-4-ylmeth­yl)cyclo­hexane-1,4-di­amine, synthesized by a condensation reaction between 1,4-cyclo­hexa­nedi­amine and 4-pyridine­carboxaldehyde according to a literature procedure (Huh & Lee, 2007 ▸). Herein we report on the crystal structure of the title salt obtained by the protonation of both amine groups in this mol­ecule.
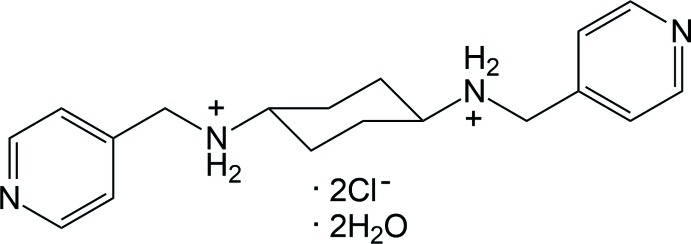


## Structural commentary   

**Figure 1:**
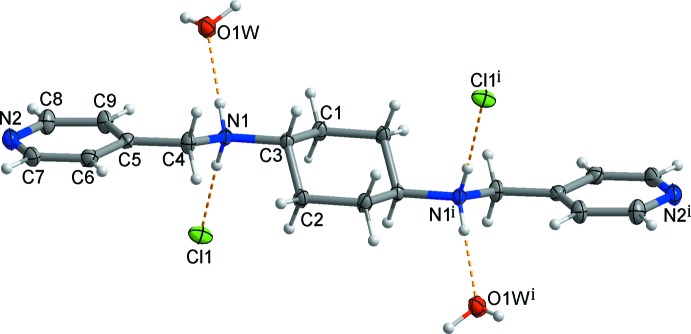
A view of the mol­ecular structure of the title salt with the atom-numbering scheme. Displacement ellipsoids are drawn at the 50% probability level and yellow dashed lines represent the inter­molecular N—H⋯O and N—H⋯Cl hydrogen bonds. [Symmetry code: (i) −*x* + 1, −*y* + 1, −*z* + 2.]

The asymmetric unit of the centrosymmetric title salt, C_18_H_26_N_4_^2+.^2Cl^−.^2H_2_O, comprises one half of *N*,*N*-bis­(pyridin-4-ylmeth­yl)cyclo­hexane-1,4-di­ammonium dication, a chloride anion and a solvent water mol­ecule (Fig. 1 ▸) due to the crystallographic inversion center located at the center of the cyclo­hexyl ring. The central cyclo­hexyl moiety of the dication adopts a chair conformation. The two *trans*-(4-pyridine)–CH_2_–NH_2_– moieties at the 1- and 4-positions of the central cyclo­hexyl ring occupy equatorial positions. The terminal pyridine ring is tilted by 27.98 (5)° with respect to the mean plane through the central cyclo­hexyl moiety (r.m.s. deviation = 0.2379 Å). The distance between the two terminal pyridine nitro­gen atoms in the dication is 15.864 (2) Å. This is slightly shorter than the N⋯N separation [15.970 (3) Å] in the dication ligand of a one-dimensional zigzag-like Co^II^ coordination polymer built up from alternate Co^II^ ions and the dication of the title salt (Lee & Lee, 2010 ▸).

## Supra­molecular features   

**Figure 2:**
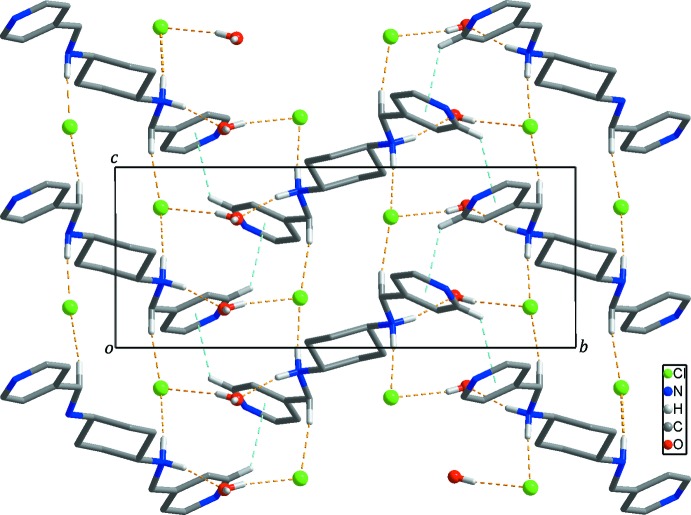
The two-dimensional undulating layer formed through inter­molecular C—H⋯π inter­actions (light-blue dashed lines) and N—H⋯O/Cl and C—H⋯Cl hydrogen bonds (yellow dashed lines). H atoms not involved in inter­molecular inter­actions have been omitted for clarity.

**Figure 3:**
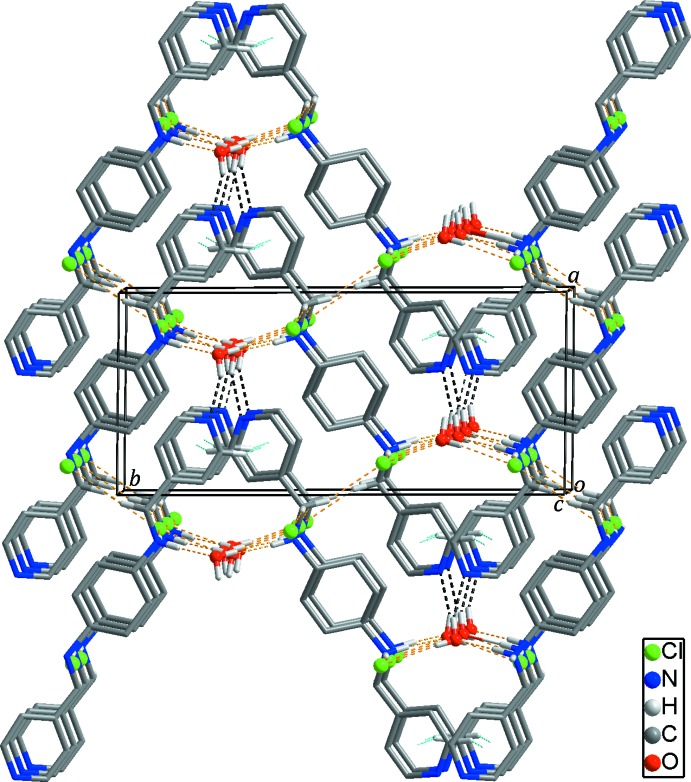
The three-dimensional supra­molecular network formed through inter­molecular N—H⋯O hydrogen bonds (black dashed lines). Inter­molecular C—H⋯π inter­actions, and N—H⋯O/Cl and C—H⋯Cl hydrogen bonds within the two-dimensional undulating layer are shown as light-blue and yellow dashed lines, respectively. H atoms not involved in inter­molecular inter­actions have been omitted for clarity.

In the crystal, adjacent dications are linked by weak C—H⋯π inter­actions, Table 1 ▸ (light-blue dashed lines in Figs. 2 ▸ and 3 ▸), resulting in the formation of a two-dimensional undulating layer-like structure extending parallel to the *bc* plane. The undulating layer is further stabilized by N—H⋯O/Cl and C—H⋯Cl hydrogen bonds (yellow dashed lines in Fig. 2 ▸) between the dications and the solvent water mol­ecules/chloride anions, respectively. Furthermore, neighboring undulating layers are connected through O—H⋯N hydrogen bonds (black dashed lines in Fig. 3 ▸) between the solvent water mol­ecules and the pyridine nitro­gen atoms, forming a three-dimensional supra­molecular network. In addition, O—H⋯Cl hydrogen bonds (Fig. 1 ▸ and Table 1 ▸) between the solvent water mol­ecules and the chloride anions are also found in the crystal.

## Synthesis and crystallization   

2 *M* hydro­chloric acid in ethanol was added to an ethanol solution of *N*,*N*-bis­(pyridin-4-yl­methyl­ene)cyclo­hexane-1,4-di­amine, synthesized according to a literature method (Huh & Lee, 2007 ▸), until pH = 4-5. The resulting mixture was left to evaporate slowly over several days, resulting in the formation of X-ray quality single crystals of the title salt.

## Refinement   

Crystal data, data collection and structure refinement details are summarized in Table 2 ▸. All C-bound H atoms were positioned geometrically with *d*(C–H) = 0.95 Å for C*sp* ^2^—H, 0.99 Å for methyl­ene, 1.00 Å for methine H atoms, and were refined as riding with *U*_iso_(H) = 1.2*U*_eq_(C). The N- and O-bound H atoms involved in hydrogen bonding were located in difference Fourier maps and refined freely [N—H = 0.878 (18) and 0.952 (17) Å; O—H = 0.78 (2) and 0.86 (2) Å].

## Supplementary Material

Crystal structure: contains datablock(s) I, New_Global_Publ_Block. DOI: 10.1107/S2056989016014626/sj5507sup1.cif

Structure factors: contains datablock(s) I. DOI: 10.1107/S2056989016014626/sj5507Isup2.hkl

CCDC reference: 1504428

Additional supporting information: crystallographic information; 3D view; checkCIF report

## Figures and Tables

**Table 1:** Hydrogen-bond geometry (Å, °) *Cg*1 is the centroid of the N2/C5–C9 ring.

*D*—H⋯*A*	*D*—H	H⋯*A*	*D*⋯*A*	*D*—H⋯*A*
N1—H1*NA*⋯O1*W*	0.878 (18)	1.881 (18)	2.7456 (15)	168.1 (16)
N1—H1*NB*⋯Cl1	0.952 (17)	2.167 (18)	3.1166 (11)	174.8 (13)
C4—H4*A*⋯Cl1^i^	0.99	2.64	3.6133 (13)	168
C4—H4*B*⋯Cl1^ii^	0.99	2.64	3.5788 (13)	158
O1*W*—H1*WA*⋯Cl1^iii^	0.78 (2)	2.37 (2)	3.1444 (11)	170.8 (18)
O1*W*—H1*WB*⋯N2^iv^	0.86 (2)	1.99 (2)	2.8242 (15)	161 (2)
C8—H8⋯*Cg*1^v^	0.95	2.74	3.3882 (15)	126

Symmetry codes: (i) 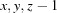; (ii) 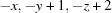; (iii) 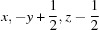; (iv) 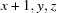; (v) 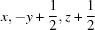.

**Table 2:** Experimental details

Crystal data	
Chemical formula	C_18_H_26_N_4_^2+^·2Cl^−^·2H_2_O
*M*_r_	405.36
Crystal system, space group	Monoclinic, *P*2_1_/*c*
Temperature (K)	173
*a*, *b*, *c* (Å)	8.2739 (2), 17.4955 (5), 7.2365 (2)
β (°)	108.756 (1)
*V* (Å^3^)	991.90 (5)
*Z*	2
Radiation type	Mo *K*α
μ (mm^−1^)	0.35
Crystal size (mm)	0.45 × 0.38 × 0.28

Data collection	
Diffractometer	Bruker APEXII CCD
Absorption correction	Multi-scan (*SADABS*; Bruker 2013 ▸)
*T*_min_, *T*_max_	0.663, 0.746
No. of measured, independent and observed [*I* > 2σ(*I*)] reflections	9616, 2475, 2199
*R*_int_	0.026
(sin θ/λ)_max_ (Å^−1^)	0.669

Refinement	
*R*[*F* ^2^ > 2σ(*F* ^2^)], *wR*(*F* ^2^), *S*	0.034, 0.088, 1.04
No. of reflections	2475
No. of parameters	134
H-atom treatment	H atoms treated by a mixture of independent and constrained refinement
Δρ_max_, Δρ_min_ (e Å^−3^)	0.33, −0.28

Computer programs: *APEX2* and *SAINT* (Bruker, 2013 ▸), *SHELXS97* and *SHELXTL* (Sheldrick, 2008 ▸), *SHELXL2014* (Sheldrick, 2015 ▸) and *DIAMOND* (Brandenburg, 2010 ▸).

## supplementary crystallographic information

### Crystal data

**Table:**

C_18_H_26_N_4_^2+^·2(Cl^−^)·2H_2_O	*F*(000) = 432
*M**_r_* = 405.36	*D*_x_ = 1.357 Mg m^−^^3^
Monoclinic, *P*2_1_/*c*	Mo *K*α radiation, λ = 0.71073 Å
*a* = 8.2739 (2) Å	Cell parameters from 4837 reflections
*b* = 17.4955 (5) Å	θ = 2.6–28.3°
*c* = 7.2365 (2) Å	µ = 0.35 mm^−^^1^
β = 108.756 (1)°	*T* = 173 K
*V* = 991.90 (5) Å^3^	Block, colourless
*Z* = 2	0.45 × 0.38 × 0.28 mm

### Data collection

**Table:**

Bruker APEXII CCD diffractometer	2199 reflections with *I* > 2σ(*I*)
φ and ω scans	*R*_int_ = 0.026
Absorption correction: multi-scan (SADABS; Bruker 2013)	θ_max_ = 28.4°, θ_min_ = 2.6°
*T*_min_ = 0.663, *T*_max_ = 0.746	*h* = −10→11
9616 measured reflections	*k* = −23→18
2475 independent reflections	*l* = −7→9

### Refinement

**Table:**

Refinement on *F*^2^	0 restraints
Least-squares matrix: full	Hydrogen site location: mixed
*R*[*F*^2^ > 2σ(*F*^2^)] = 0.034	H atoms treated by a mixture of independent and constrained refinement
*wR*(*F*^2^) = 0.088	*w* = 1/[σ^2^(*F*_o_^2^) + (0.0443*P*)^2^ + 0.3432*P*] where *P* = (*F*_o_^2^ + 2*F*_c_^2^)/3
*S* = 1.04	(Δ/σ)_max_ < 0.001
2475 reflections	Δρ_max_ = 0.33 e Å^−^^3^
134 parameters	Δρ_min_ = −0.28 e Å^−^^3^

### Special details

**Table:**

Geometry. All esds (except the esd in the dihedral angle between two l.s. planes) are estimated using the full covariance matrix. The cell esds are taken into account individually in the estimation of esds in distances, angles and torsion angles; correlations between esds in cell parameters are only used when they are defined by crystal symmetry. An approximate (isotropic) treatment of cell esds is used for estimating esds involving l.s. planes.

### Fractional atomic coordinates and isotropic or equivalent isotropic displacement parameters (Å^2^)

**Table:**

	*x*	*y*	*z*	*U*_iso_*/*U*_eq_	
Cl1	0.16235 (4)	0.40135 (2)	1.27614 (5)	0.02515 (11)	
N1	0.21335 (12)	0.39798 (6)	0.86785 (16)	0.0163 (2)	
H1NA	0.243 (2)	0.3529 (10)	0.836 (2)	0.029 (4)*	
H1NB	0.198 (2)	0.3956 (9)	0.993 (3)	0.027 (4)*	
N2	−0.38725 (13)	0.28164 (7)	0.67407 (17)	0.0249 (3)	
C1	0.48104 (14)	0.58254 (7)	0.96734 (19)	0.0195 (3)	
H1A	0.5016	0.5882	0.8406	0.023*	
H1B	0.4582	0.6339	1.0105	0.023*	
C2	0.32520 (14)	0.53131 (7)	0.94108 (19)	0.0193 (3)	
H2A	0.2981	0.5292	1.0647	0.023*	
H2B	0.2254	0.5530	0.8390	0.023*	
C3	0.36023 (14)	0.45089 (7)	0.88272 (17)	0.0162 (2)	
H3	0.3791	0.4532	0.7531	0.019*	
C4	0.05169 (14)	0.42138 (7)	0.71776 (18)	0.0195 (3)	
H4A	0.0688	0.4217	0.5885	0.023*	
H4B	0.0233	0.4741	0.7460	0.023*	
C5	−0.09626 (14)	0.36983 (7)	0.70865 (17)	0.0172 (2)	
C6	−0.25944 (15)	0.39624 (7)	0.60508 (18)	0.0202 (3)	
H6	−0.2743	0.4451	0.5446	0.024*	
C7	−0.39921 (15)	0.35070 (8)	0.59139 (19)	0.0228 (3)	
H7	−0.5094	0.3694	0.5196	0.027*	
C8	−0.23028 (16)	0.25662 (8)	0.7712 (2)	0.0259 (3)	
H8	−0.2191	0.2073	0.8291	0.031*	
C9	−0.08246 (15)	0.29821 (8)	0.79263 (19)	0.0221 (3)	
H9	0.0261	0.2778	0.8638	0.027*	
O1W	0.28682 (13)	0.26372 (6)	0.71524 (16)	0.0265 (2)	
H1WA	0.263 (2)	0.2234 (13)	0.743 (3)	0.043 (6)*	
H1WB	0.388 (3)	0.2576 (12)	0.707 (3)	0.056 (6)*	

### Atomic displacement parameters (Å^2^)

**Table:**

	*U*^11^	*U*^22^	*U*^33^	*U*^12^	*U*^13^	*U*^23^
Cl1	0.02915 (18)	0.02300 (19)	0.02559 (18)	0.00706 (12)	0.01200 (13)	0.00199 (12)
N1	0.0129 (4)	0.0159 (5)	0.0193 (5)	−0.0018 (4)	0.0040 (4)	−0.0008 (4)
N2	0.0176 (5)	0.0311 (6)	0.0261 (6)	−0.0060 (4)	0.0073 (4)	−0.0029 (5)
C1	0.0153 (5)	0.0144 (6)	0.0260 (6)	−0.0004 (4)	0.0027 (5)	−0.0002 (5)
C2	0.0131 (5)	0.0156 (6)	0.0275 (6)	0.0000 (4)	0.0042 (4)	−0.0017 (5)
C3	0.0129 (5)	0.0158 (6)	0.0197 (6)	−0.0025 (4)	0.0049 (4)	−0.0009 (4)
C4	0.0137 (5)	0.0208 (6)	0.0214 (6)	−0.0016 (4)	0.0021 (4)	0.0027 (5)
C5	0.0154 (5)	0.0204 (6)	0.0161 (5)	−0.0022 (4)	0.0052 (4)	−0.0044 (5)
C6	0.0186 (6)	0.0201 (6)	0.0202 (6)	0.0007 (5)	0.0040 (4)	−0.0024 (5)
C7	0.0147 (5)	0.0288 (7)	0.0237 (6)	0.0005 (5)	0.0044 (4)	−0.0051 (5)
C8	0.0226 (6)	0.0269 (7)	0.0272 (7)	−0.0053 (5)	0.0068 (5)	0.0041 (5)
C9	0.0158 (5)	0.0251 (7)	0.0234 (6)	−0.0015 (5)	0.0034 (5)	0.0030 (5)
O1W	0.0232 (5)	0.0182 (5)	0.0418 (6)	−0.0017 (4)	0.0155 (4)	0.0001 (4)

### Geometric parameters (Å, º)

**Table:**

N1—C4	1.4839 (15)	C3—H3	1.0000
N1—C3	1.5037 (14)	C4—C5	1.5047 (16)
N1—H1NA	0.878 (18)	C4—H4A	0.9900
N1—H1NB	0.952 (17)	C4—H4B	0.9900
N2—C8	1.3361 (17)	C5—C9	1.3812 (18)
N2—C7	1.3378 (18)	C5—C6	1.3955 (16)
C1—C3^i^	1.5257 (16)	C6—C7	1.3814 (17)
C1—C2	1.5311 (16)	C6—H6	0.9500
C1—H1A	0.9900	C7—H7	0.9500
C1—H1B	0.9900	C8—C9	1.3883 (17)
C2—C3	1.5234 (17)	C8—H8	0.9500
C2—H2A	0.9900	C9—H9	0.9500
C2—H2B	0.9900	O1W—H1WA	0.78 (2)
C3—C1^i^	1.5257 (16)	O1W—H1WB	0.86 (2)
			
C4—N1—C3	113.58 (9)	C1^i^—C3—H3	108.9
C4—N1—H1NA	108.5 (11)	N1—C4—C5	113.32 (10)
C3—N1—H1NA	106.7 (11)	N1—C4—H4A	108.9
C4—N1—H1NB	110.1 (10)	C5—C4—H4A	108.9
C3—N1—H1NB	108.0 (10)	N1—C4—H4B	108.9
H1NA—N1—H1NB	109.9 (14)	C5—C4—H4B	108.9
C8—N2—C7	116.79 (11)	H4A—C4—H4B	107.7
C3^i^—C1—C2	111.21 (10)	C9—C5—C6	117.74 (11)
C3^i^—C1—H1A	109.4	C9—C5—C4	124.99 (11)
C2—C1—H1A	109.4	C6—C5—C4	117.26 (11)
C3^i^—C1—H1B	109.4	C7—C6—C5	119.38 (12)
C2—C1—H1B	109.4	C7—C6—H6	120.3
H1A—C1—H1B	108.0	C5—C6—H6	120.3
C3—C2—C1	110.33 (9)	N2—C7—C6	123.30 (12)
C3—C2—H2A	109.6	N2—C7—H7	118.3
C1—C2—H2A	109.6	C6—C7—H7	118.3
C3—C2—H2B	109.6	N2—C8—C9	124.02 (13)
C1—C2—H2B	109.6	N2—C8—H8	118.0
H2A—C2—H2B	108.1	C9—C8—H8	118.0
N1—C3—C2	111.53 (9)	C5—C9—C8	118.76 (12)
N1—C3—C1^i^	107.82 (9)	C5—C9—H9	120.6
C2—C3—C1^i^	110.72 (10)	C8—C9—H9	120.6
N1—C3—H3	108.9	H1WA—O1W—H1WB	104 (2)
C2—C3—H3	108.9		
			
C3^i^—C1—C2—C3	−56.73 (15)	C9—C5—C6—C7	0.35 (18)
C4—N1—C3—C2	61.58 (13)	C4—C5—C6—C7	179.52 (12)
C4—N1—C3—C1^i^	−176.66 (10)	C8—N2—C7—C6	−1.06 (19)
C1—C2—C3—N1	176.51 (10)	C5—C6—C7—N2	0.4 (2)
C1—C2—C3—C1^i^	56.44 (15)	C7—N2—C8—C9	1.0 (2)
C3—N1—C4—C5	−177.89 (10)	C6—C5—C9—C8	−0.41 (19)
N1—C4—C5—C9	−14.96 (18)	C4—C5—C9—C8	−179.51 (12)
N1—C4—C5—C6	165.94 (11)	N2—C8—C9—C5	−0.3 (2)

Symmetry code: (i) −*x*+1, −*y*+1, −*z*+2.

### Hydrogen-bond geometry (Å, º)

*Cg*1 is the centroid of the N2/C5–C9 ring.

**Table:**

*D*—H···*A*	*D*—H	H···*A*	*D*···*A*	*D*—H···*A*
N1—H1*NA*···O1*W*	0.878 (18)	1.881 (18)	2.7456 (15)	168.1 (16)
N1—H1*NB*···Cl1	0.952 (17)	2.167 (18)	3.1166 (11)	174.8 (13)
C4—H4*A*···Cl1^ii^	0.99	2.64	3.6133 (13)	168
C4—H4*B*···Cl1^iii^	0.99	2.64	3.5788 (13)	158
O1*W*—H1*WA*···Cl1^iv^	0.78 (2)	2.37 (2)	3.1444 (11)	170.8 (18)
O1*W*—H1*WB*···N2^v^	0.86 (2)	1.99 (2)	2.8242 (15)	161 (2)
C8—H8···*Cg*1^vi^	0.95	2.74	3.3882 (15)	126

Symmetry codes: (ii) *x*, *y*, *z*−1; (iii) −*x*, −*y*+1, −*z*+2; (iv) *x*, −*y*+1/2, *z*−1/2; (v) *x*+1, *y*, *z*; (vi) *x*, −*y*+1/2, *z*+1/2.
